# Hot electron induced non-saturation current behavior at high electric field in InAlN/GaN heterostructures with ultrathin barrier

**DOI:** 10.1038/srep37415

**Published:** 2016-11-23

**Authors:** Lei Guo, Xuelin Yang, Anqi Hu, Zhihong Feng, Yuanjie Lv, Jie Zhang, Jianpeng Cheng, Ning Tang, Xinqiang Wang, Weikun Ge, Bo Shen

**Affiliations:** 1 State Key Laboratory of Artificial Microstructure and Mesoscopic Physics, School of Physics, Peking University, Beijing 100871, China; 2 National Key Laboratory of Application Specific Integrated Circuit, Hebei Semiconductor Research Institute, Shijiazhuang 050051, China; 3 Collaborative Innovation Center of Quantum Matter, Beijing 100871, China

## Abstract

The high-field transport characteristics of nearly lattice-matched InAlN/GaN heterostructures with different barrier thickness were investigated. It is found that the current in the InAlN/GaN heterostructures with ultrathin barrier shows unsaturated behaviors (or secondary rising) at high voltage, which is different from that of AlGaN/GaN heterostructures. This phenomenon is more obvious if the barrier thickness is thinner and the channel width is narrower. The experimental results demonstrate that it is the increasing carrier density excited from the more defect states by the hot electrons with larger electron saturation velocity that results in the unsaturated current behaviors in InAlN/GaN heterostructures. Our results pave a way for further optimizing InAlN barrier design and improving the reliability of InAlN/GaN HEMTs.

GaN-based high electron mobility transistors (HEMTs) are promising devices for next generation high frequency and high power applications^1^,^2^. In particular, InAlN/GaN HEMTs are excellent alternatives to AlGaN/GaN HEMTs for ultrahigh frequency device applications. The current gain cutoff frequency of ultrascaled InAlN/GaN HEMTs has already been up to 400 GHz, as reported recently^3^,^4^,^5^. However, several questions related to the device reliability at high electric field are still open for InAlN/GaN HEMTs. On one hand, compared to AlGaN, the material quality of InAlN still need to be improved due to the different optimized growth conditions of InN and AlN and the expected large immiscibility gap of the ternary alloy. Thus, there are large density of defects which may correlate to the problem of more serious degradation of the InAlN/GaN HEMTs^6^,^7^,^8^. On the other hand, the larger spontaneous polarization in InAlN/GaN heterostructures contributes to much higher two-dimensional electron gas (2DEG) density in the lattice-matched In_0.18_Al_0.82_N/GaN system with thin barrier layer^9^,^10^. Some studies have proved that the higher electron density of InAlN/GaN may result in lower hot phonon (HP) lifetime and weaker HP effect, and thus lead to higher electron velocity and larger kinetic energy than that of AlGaN/GaN heterostructure^11^,^12^,^13^,^14^. Although the higher electron velocity is benefit to high frequency devices, the hot-electron temperature (*T*_*e*_) in InAlN/GaN is much higher than that in AlGaN/GaN. For example, Kuzmik *et al.* reported that *T*_*e*_ in InAlN/GaN may reach as high as 30000 K^6^,^15^. Considering the hot electrons with higher energy and facing larger defects density, the transport characteristics of InAlN/GaN at high electric field are more complex and lack of systematic studies, compared with that of AlGaN/GaN. Especially, the behavior of the current at high filed in InAlN/GaN is quite different from that in AlGaN/GaN, the saturation tendency of the current observed in the latter cannot be observed again in InAlN/GaN heterostructures^16^. Detailed mechanism about this phenomenon is yet to be fully understood, although it is important for further improving the reliability of InAlN/GaN HEMTs. In this work, we investigate the high-field transport characteristics of In_0.18_Al_0.82_N/GaN with different InAlN barrier thickness. Different from that of AlGaN/GaN, the current in the InAlN/GaN heterostructure with ultrathin barrier shows obvious secondary rising after a saturation region at higher voltage. It is demonstrated that the phenomenon is related with the larger electron kinetic energy as well as the larger density of defects. In fact, it is the increased carrier density excited from the defect states by hotter electrons leads to the secondary rising current in the InAlN/GaN heterostructure.

Table 1 shows the sample information including barrier thickness, electron mobility (*μ*) and sheet density (*n*) measured at room temperature by a Hall Effect measurement system with Van der Pauw method. With the thickness of the InAlN barrier increasing from 4 to 100 nm, the electron sheet density increases from 1.75 × 10^13^ to 2.60 × 10^13^ cm^−2^, and the mobility decreases from 1500 to 790 cm^2^/Vs. Figure 1 gives the schematic H-shaped test structure. The current-voltage (I-V) curves of the AlGaN/GaN heterostructure shown in the Fig. 2 are the typical characteristics for GaN-based material system^17^. When the applied voltage changing from low to high values, there appear three regions that show different relationships between current and voltage. At the low electric field region, there is a linear relation where the Ohm’s law is obeyed, and the current linearly relies on the voltage. With further increasing voltage, the relation becomes sublinear, which is due to the fact that the electron energy is gradually enhanced and so that more serious scattering is induced, leading to electron mobility decrease and a sublinear dependence between current and voltage. Finally, when the voltage is above 80 V (about 80 kV/cm for the electric field), the current gradually rises into the saturation region, the saturation velocity (*v*_*sat*_) can be inferred from current density:


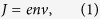


where *e* is the charge of electron, *n* is the sheet density, and *v* is the electron drift mobility. According to equation (1), the saturation velocity is calculated to be about 1.3 × 10^7^ cm/s, as shown in Fig. 3. It should be mentioned that the saturation velocity *v*_*sat*_ is theoretically determined by the longitudinal optical (LO) phonon and should be of the order of:


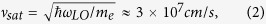


where *ħω*_*LO*_ is the energy of the LO phonon, and *m*_*e*_ is the electron effective mass. However, the measured *v*_*sat*_ in GaN is much lower than the theoretical value, which is mainly caused by the HP effect due to the large number of hot phonons. It is noticed that the LO phonon lifetime (~0.35–2.5 ps) is much longer than the spontaneous LO phonon emission time (~ 10 fs), so that there will be a great accumulation of phonons in the system. The accumulated none-equilibrium LO phonons emitted by hot electrons can reduce the energy relaxation rate and enhance the momentum relaxation rate of the hot electrons. Thus, *v*_*sat*_ is much lower due to more serious momentum scattering^18^,^19^. In brief, the lower *v*_*sat*_is mainly limited by the longer hot photon lifetime.

As shown in Fig. 2, the I-V characteristics of a 100 nm-thick barrier InAlN/GaN is similar with that of AlGaN/GaN heterostructure, and the current gradually increases to saturation with increasing voltage. However, as the InAlN barrier thickness decreases to 8 nm, it is interesting to find that the current shows a secondary slightly rising behavior after saturation with further increasing voltage. The phenomenon of the secondary rising at sufficiently high voltage is more obvious when the InAlN barrier thickness further decreases to 4 nm. For 4 nm-thick barrier InAlN/GaN, with voltage increasing from 6 V to 80 V, the I-V characteristic changes from linear to sublinear. Further increasing the voltage from 80 V to 260 V, the current basically keeps the saturation value due to the velocity saturation. The *v*_*sat*_ inferred from the saturation current in the InAlN/GaN heterostructure with 4 nm-thick-InAlN barrier is about 2.3 × 10^7^ cm/s (shown in Fig. 3) which is much higher than that in AlGaN/GaN heterostructures. However when the voltage is above a critical voltage, the current shows a surprising secondary rising with the voltage increasing from 270 V to 360 V, and no saturation tendency is observed. It should be mentioned that the current density rising could be due to the increasing of either carrier sheet density or velocity, according to equation (1). At the low voltage region (linear and sublinear region), the rising of current is due to the increased velocity. But that is not the cause for the secondary rising at the high voltage range, since the saturation velocity is determined by the energy of the LO phonon as mentioned above, being independent on the voltage. In addition, if the carrier density is constant and independent on the voltage, the inferred velocity at 360 V would be up to about 3.3 × 10^7^ cm/s which is much higher over the theoretical upper limit value, hence not possible. Therefore, the secondary rising of the current should mainly result from the increased carrier density induced by the interaction between the hot electrons and material defects.

To further understand the above phenomenon, there are two questions need to be addressed at first: (i) why does the secondary rising happen in InAlN/GaN but not in AlGaN/GaN heterostructures, despite that they have the same GaN channel; (ii) why is the phenomenon more obvious in the sample with 4 nm barrier than that with thicker barrier? We can find answers from the view point of hot electron behaviors and material defects.

Firstly, the value of *v*_*sat*_ for the hot electron as a function of the carrier density was extracted (Fig. 3) according to equation (1) for different samples. With the electron density increasing from 8.0 × 10^12^ cm^−2^ in AlGaN/GaN heterostructure to 17.5 × 10^12^ cm^−2^ in the InAlN/GaN heterostructure with 4 nm-InAlN barrier, the value of *v*_*sat*_ increases from 1.3 × 10^7^ cm/s to 2.3 × 10^7^ cm/s, reaching a highest value. Then, with the electron density further increasing to 23.5 × 10^12^ cm^−2^ in the 8 nm-InAlN barrier sample and finally increasing to 26.0 × 10^12^ cm^−2^ in the 100-nm InAlN barrier sample, *v*_*sat*_ gradually decreases down to 0.9 × 10^7^ cm/s. As discussed above, *v*_*sat*_ is mainly related with the HP lifetime, the variation pattern of *v*_*sat*_ is consistent with that of the HP lifetime as previous reported: the HP lifetime is closely related to the electron density due to plasma-LO phonon coupling, the estimated resonant density is about 7.0 × 10^12^ cm^−2^ and shifts to higher values with increasing the applied power^13^,^20^. When the electron density is below that value, the HP lifetime will decrease with increasing density and thus *v*_*sat*_ will increase, which is consistent with the results in the present work referring to the sample change from AlGaN/GaN heterostructure to InAlN/GaN heterostructure with 4 nm-barrier. The case is converse when the density is beyond that value: the HP lifetime will increase with increasing density and thus *v*_*sat*_ will decrease, that can perfectly explain the results of InAlN/GaN with different barrier thickness.

The temperature-dependence of the 2DEG sheet density of the InAlN/GaN heterostructure (4 nm-barrier) and AlGaN/GaN heterostructure are shown in Fig. 4. In the temperature range of 100–700 K, the carrier density of AlGaN/GaN has a weak dependence on temperature, showing a typical 2DEG behavior. However, the temperature-dependence of the density in the InAlN/GaN heterostructure shows a standard shallow-donor behavior. The activation energy of the donor is around 90 meV from the fitting result using a donor model. Since the GaN buffer is the same between the AlGaN/GaN and InAlN/GaN heterostructure, the donor may exist in the InAlN layer or interface. It should be mentioned that there are also other defect states with deeper level existing in the InAlN layer or interface, as revealed by capacitance-voltage and deep-level transient spectroscopy experiments^8^,^21^,^22^,^23^. The donor defect with an activation energy of 90 meV exhibited by the temperature-dependence Hall measurement is just the most easily excited by the high temperature and contributes to the sheet density. In fact, the hot electron temperature in the InAlN/GaN heterostructure may be much higher than the upper limitation of the temperature range in our Hall testing system. Therefore, it is reasonable to deduce that the hot electrons can also easily excite the electrons trapped in the deeper defects states in the InAlN/GaN heterostructure, which may also contributes to the secondary rising behaviors.

Now, considering interaction between the hot electrons and material defects, it is easier to understand that the electrons trapped in these donor states are excited to conduction band by hot electron at high voltage and result in the secondary rising current in InAlN/GaN rather than AlGaN/GaN. Moreover, higher *v*_*sat*_ in InAlN/GaN with lower carrier density corresponds to much larger kinetic energy which can more easily excite trapped electron into conduction band. Thus, the secondary rising current phenomenon is more obvious in the InAlN/GaN with 4 nm-barrier than that with thicker ones.

To further confirm our explanation, we prepared several test devices of the H-shaped geometry with different channel widths changing from 5 to 20 *μm* for InAlN/GaN heterostructures with 4 nm-barrier. Our previous work has confirmed that narrower channel can enhance the momentum relaxation of LO phonons and lower the lifetime of HP, and thus help to increase *v*_*sat*_^24^. According to this result, the hot electrons in a narrower channel have larger kinetic energy than that in a wider channel, and thus more easily excite the electrons trapped in defect states. Figure 5 shows the I-V characteristics of the devices with different channel widths. We can see that the current of the test device with 5 *μm* width channel shows a larger secondary rising at a smaller voltage than that of devices with 15 and 20 *μm* width channels. This result further confirms that the interaction between the hot electrons with large kinetic energy and the large density of defects are the main reason of the secondary rising current.

In summary, we have investigated the high-field transport properties of InAlN/GaN heterostructures. Different from that of AlGaN/GaN, the current of InAlN/GaN heterostructure shows a secondary rising at higher voltage after a saturation region. The secondary rising phenomenon is more obvious in samples with thinner barrier thickness and narrower channel width. These behaviors are attributed to the larger electron kinetic energy and more defects in InAlN/GaN than that in AlGaN/GaN heterostructures. It is the increasing carrier density excited from the defect states by hot electrons that results in the secondary rising current in InAlN/GaN heterostructures with ultrathin barrier. The present work highlights the importance of InAlN barrier thickness as well as the quality for improving the reliability of InAlN/GaN HEMTs.

## Methods

### Sample Preparation

**Figure 1:**
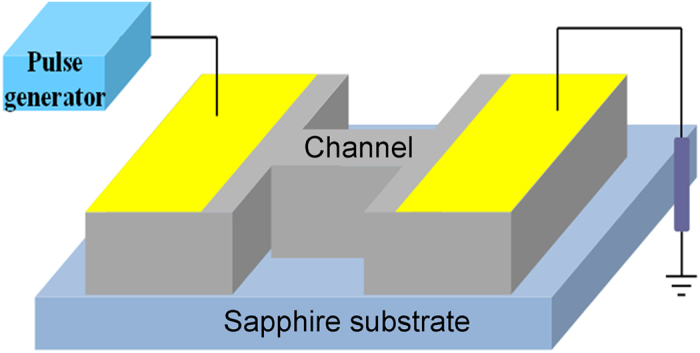
The schematic H-shaped test structure used for the measurements.

The In_0.18_Al_0.82_N/GaN and Al_0.24_Ga_0.76_N/GaN heterostructures used in this work were grown on *c*-plane sapphire substrate by metal-organic chemical vapor deposition (MOCVD). The heterostructures consisted of a 2.5 *μm* thick GaN buffer layer, a thin AlN spacer, and In_0.18_Al_0.82_N or Al_0.24_Ga_0.76_N barrier. The growth temperature for In_0.18_Al_0.82_N and Al_0.24_Ga_0.76_N is about 800 °C and 1070 °C, respectively. The channel dimensions (*L* × *W*) are 10 *μm* × 15 *μm*, and the mesa was etched down to the substrate. The sample’s mesa was fabricated with an H-shaped geometry (Fig. 1) which may (i) make the electric field in the channel homogenous, (ii) minimize the effects of contact resistance, and (iii) control the carrier injection from the Ohmic contacts^25^. The Ohmic contacts (300 *μm* × 500 *μm*) on both sides were processed by Ti/Al/Ni/Au metallization and annealed at 850 °C for 35 s. The contact resistance was 0.9 Ω · *mm* estimated at low electric fields by transmission line model (TLM). The channel at the middle was passivated with a 100 nm Si_3_N_4_ passivation layer.

### Measurements

High-field current-voltage (I-V) characteristics were obtained by nanosecond pulsed technique on H-shape sample mounted in a 50 Ω matched circuit. The voltage pulse length was fixed at 80 ns with a repetition rate of 1 Hz in order to minimize the self-heating effect.

## Additional Information

**How to cite this article**: Guo, L. *et al.* Hot electron induced non-saturation current behavior at high electric field in InAlN/GaN heterostructures with ultrathin barrier. *Sci. Rep.* **6**, 37415; doi: 10.1038/srep37415 (2016).

**Publisher's note:** Springer Nature remains neutral with regard to jurisdictional claims in published maps and institutional affiliations.

## Figures and Tables

**Figure 2:**
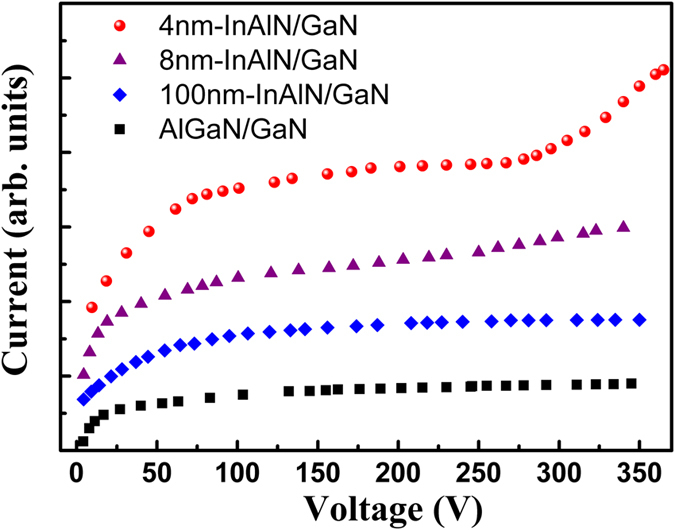
I-V characteristics of the AlGaN/GaN and InAlN/GaN heterostructures with different barrier thickness.

**Figure 3:**
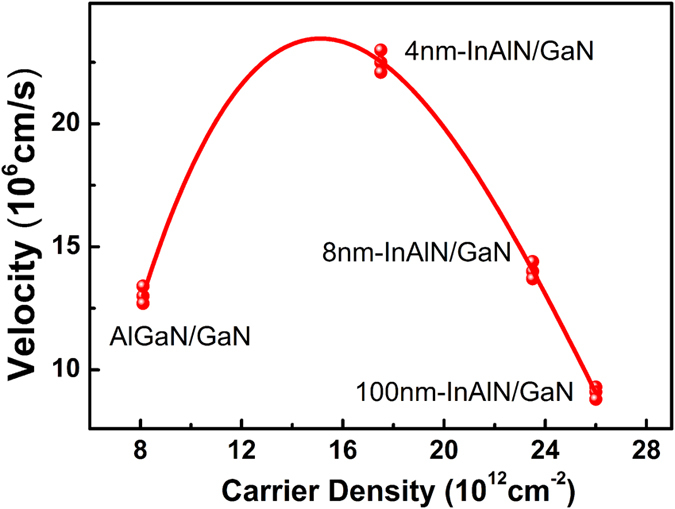
Dependence of experimental saturation velocity on carrier density for different samples shown in Table 1 . The channel dimensions (*L* × *W*) of testing samples is 10 *μm* × 15 *μm*. The scattered data represent results of different areas from the same wafer.

**Figure 4:**
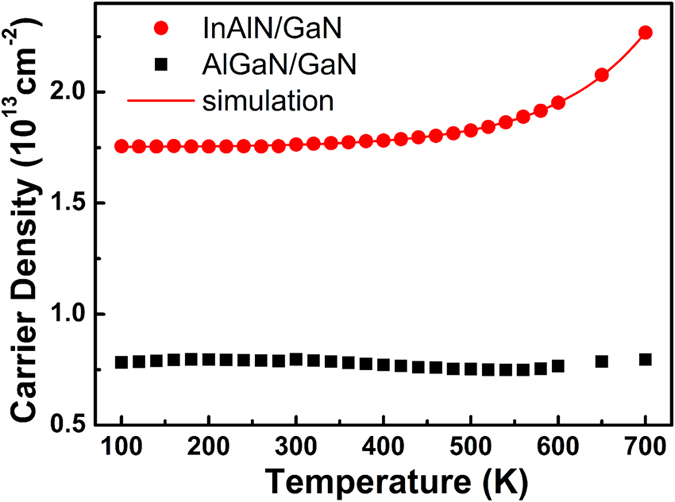
Temperature-dependence carrier density of the AlGaN/GaN heterostructure and InAlN/GaN heterostructure with 4 nm-InAlN barrier.

**Figure 5:**
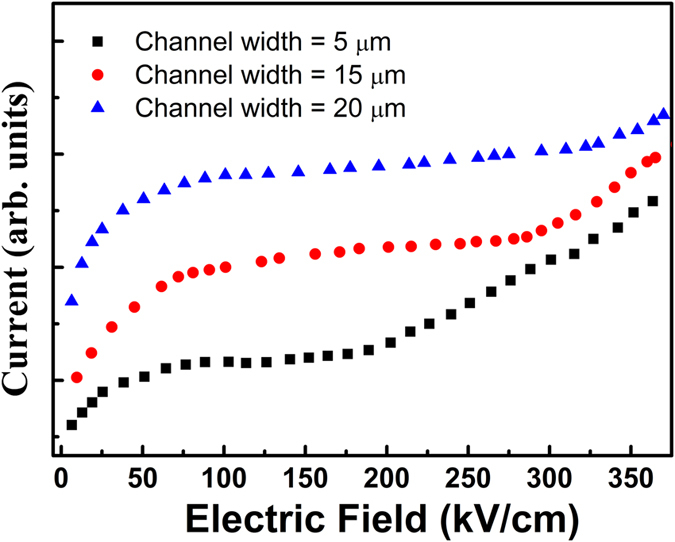
Dependence of current on electric field for the InAlN/GaN heterostructure with 4 nm-barrier. Different curves represent the characteristics for the test devices with different channel widths changing from 5 to 20 *μm*.

**Table 1:** Sample structures and electrical properties measured at room temperature.

Samples	Barrier layer	Barrier thickness (nm)	Density (×10^12^ cm^−2^)	Mobility (cm^2^/Vs)
# 1	In_0.18_Al_0.82_N	4	17.5	1500
# 2	In_0.18_Al_0.82_N	8	23.5	1400
# 3	In_0.18_Al_0.82_N	100	26.0	790
# 4	Al_0.24_Ga_0.76_N	20	8.1	2050
